# Three complete mitochondrial genomes of straw-rotting edible fungus *Volvariella volvacea* using next generation sequencing

**DOI:** 10.1080/23802359.2018.1511849

**Published:** 2018-10-27

**Authors:** Jianing Wan, Yan Li, Hong Wang, Lihua Tang, Zhengpeng Li, Chenli Zhou, Qi Tan, Dapeng Bao, Ruiheng Yang

**Affiliations:** National Engineering Research Center of Edible Fungi, Ministry of Science and Technology (MOST), Key Laboratory of Edible Fungi Resources and Utilization (South), Ministry of Agriculture, Institute of Edible Fungi, Shanghai Academy of Agricultural Sciences, Shanghai, China

**Keywords:** *Volvariella volvacea*, Agaricales, Pluteaceae, mitogenome

## Abstract

The straw-rotting edible fungus *Volvariella volvacea* is a widely cultivated edible fungus across China and Southeast Asian countries. Three complete mitochondrial genomes of *V. volvacea* from China, Thailand, and India were determined using the next-generation sequencing technology. The genome sizes of the three strains (China, Thailand, and India) were 62,541 bp, 64,531 bp, and 65,668 bp with GC contents of 38.46%, 38.56%, and 38.52%, respectively. All the genomes encoded 14 conserved protein-coding genes, the small ribosomal RNA subunits (rns), large ribosomal RNA subunits (rnl), and 23 tRNAs were located on the same strand. In the putative protein-coding genes, four introns were distributed in cox1 in the genomes of V23-1 and V8. 5 introns (four introns invaded into cox1and one intron invaded into cob) were detected in Tai8. The phylogenetic analysis confirmed that *V. volvacea* was a number of Agaricales. This mitochondrial genome may open new avenues for understanding the phylogeny and evolution of Pluteaceae and Agaricales.

*Volvariella volvacea* (Bull, ex, Fr.) Singer., the Chinese straw mushroom, is an important edible fungus cultivated extensively across China and Southeast Asian countries (Chang and Li 1991). According to traditional Chinese medicine, consuming the mushroom is good for the liver and stomach, relieves summer heats, and enriches milk production in women following childbirth (Kalava and Menon 2012; Wang et al. 2014). The genome, transcriptome, and some function genes of *V. volvacea* have been analysed in several studies (Bao et al. 2013; Chen et al. 2013, 2016; Gong et al. 2015). However, little is known about the mitochondrial genome of this fungus. In this study, we report three complete mitogenomes of *V. volvacea* and may provide a phylogenetic analysis of related taxa based on concatenated mitochondrial protein-coding genes.

Three strains provided by Institute of Edible Fungi, Shanghai Academy of Agricultural Sciences were from China, Thailand, and India and named *V. volvacea* V23-1, Tai8, and V8, respectively. All the monokaryon strains were isolated from dikaryon using the protoplast method (Bao et al. 2013) and cultivated under 25 °C on PDA medium for five days. Total DNA extraction, library construction, Illumina sequencing and sequences processing were performed according to the methods previous published (Yang et al. 2016; Xu et al. 2018). The filtered high quality sequences were assembled into different contigs using A5-miseq 2.0 (Sydney, Australia, Coil et al. 2014). And all the assembled contigs were mapped to the database of fungal mitogenomes to extract contigs belonged to the mitogenome of *V. volvacea* using Blast. The completed mitogenome was annotated using MFannot (http://megasun.bch.umontreal.ca/cgi-bin/mfannot/mfannotInterface.pl). And neighbour-joining phylogenetic analysis of 26 other species belonged to Agaricomycotina was conducted using MEGA 7.0 (Tokyo, Japan, Kumar et al. 2016) (Figure 1).

**Figure 1.**
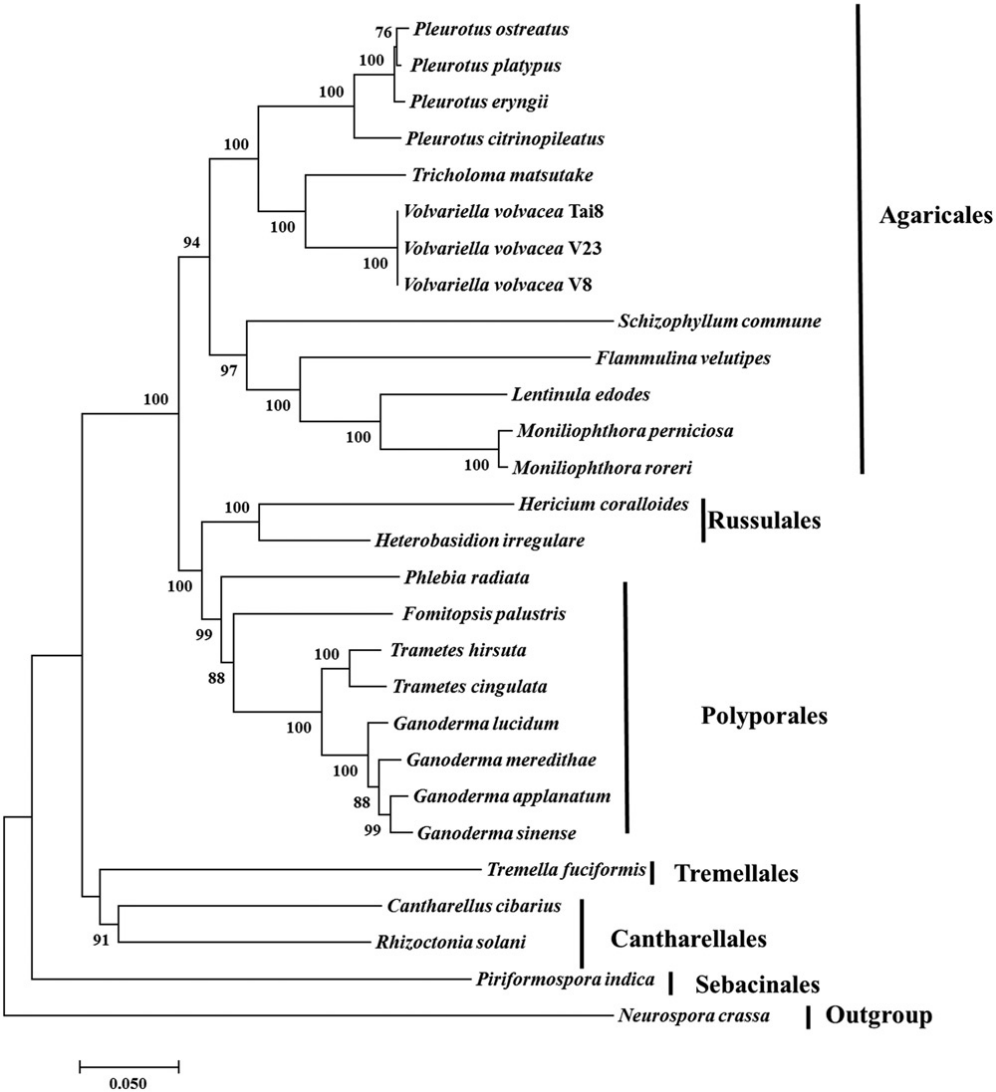
Neighbour-Joining analysis of 28 species belonging to Agaricomycotina (including three *V. volvacea* strains) based on 13 concatenated amino acid sequences. All the other 25 species used for phylogeny were listed following: *Cantharellus cibarius* (NC_020368), *Flammulina velutipes* (NC_021373), *Fomitopsis palustris* (NC_034349), *Hericium coralloides* (NC_033903), *Ganoderma applanatum* (NC_027188), *Ganoderma lucidum* (NC_021750), *Ganoderma meredithae* (NC_026782), *Ganoderma sinense* (NC_022933), *Heterobasidion irregulare* (NC_024555), *Lentinula edodes* (NC_018365), *Moniliophthora perniciosa* (NC_005927), *Moniliophthora roreri* (NC_015400), *Pleurotus citrinopileatus* (NC_036998), *Pleurotus ostreatus* (NC_009905), *Pleurotus platypus* (NC_036999), *Phlebia radiata* (NC_020148), *Rhizoctonia solani* (HF546977), *Schizophyllum commune* (NC_003049), *Serendipita indica* (FQ859090), *Trametes hirsuta* (NC_037239), *Tremella fuciformis* (NC_036422), *Trichosporon asahii* var. asahii (MT: JH925097), *Trametes cingulata* (NC_013933), and *Tricholoma matsutake* (NC_028135). *Neurospora crassa* (NC_026614) was served as outgroup. The percentages of replicate trees in which the associated taxa clustered together in the bootstrap test (1000 replicates) were shown next to the branches.

A total of 6232M bp, 1197M bp, and 7790M bp clean data were generated from the sequencing platform for the strains V23-1, Tai8, and V8, respectively. The three circus mitogenomes of V23-1, Tai8, and V8 were 62,541 bp, 64,531 bp, and 65,668 bp in length with GC contents of 38.46%, 38.56%, and 38.52%, respectively. Gene predictions showed 54, 55, and 56 genes determined in V23-1, Tai8, and V8, respectively. Among these genes, 14 conserved protein-coding genes, one small ribosomal RNA subunits (rns) and one large ribosomal RNA subunits (rnl) were detected in all the genomes. The 14 conserved protein-coding genes encoded the three ATP synthases (atp6, apt 8, and apt 9), one apocytochrome b (cob), three cytochrome oxidases (cox1–3), seven subunits of NAD dehydrogenase (nad1-6 and nad4L). Fifteen, 16, and 16 hypothical genes were predicted in V23-1, Tai8, and V8, respectively. The set of 23 tRNA genes could code for all 20 standard amino acids in all the genomes. The four introns were distributed in cox1 but no intron was found in other 13 genes in strains V23-1 and V8. In Tai8, besides four introns invaded into cox1, one intron was detected in cob. Some studies revealed that introns were the main contributors to mitochondrial genome size variations among different strains (Zhang et al. 2015).

A total of 13 amino acid sequences were for phylogenetic analysis, including atp6, atp8, atp9, cob, cox1, cox2, cox3, nad1, nad3, nad4, nad4L, nad5, and nad6. The concatenated sequences were aligned using Clustal (Thompson et al. 2010). Phylogenetic relationship based on concatenated protein sequences confirmed that *V. volvacea* was a number of Agaricales and all the three genomes clustered together. *Volvariella volvacea* clustered together with *Tricholoma matsutake* belonged to Tricholomataceae. The evolutionarily relationship among Agaricales, Russulales, Polyporales, Cantharellales, and Sebacinales was in accordance with results of previously study (Matheny et al. 2006; Garcia-Sandoval et al. 2011; Zhao et al. 2017). The mitogenomes of *V. volvacea* would provide new insights into understanding the phylogeny and evolution of Pluteaceae and Agaricales.
